# The psychometric properties of PHQ-4 anxiety and depression screening scale among out of school adolescent girls and young women in Tanzania: a cross-sectional study

**DOI:** 10.1186/s12888-020-02735-5

**Published:** 2020-06-19

**Authors:** Jacqueline Materu, Evodius Kuringe, Daniel Nyato, Anthony Galishi, Amasha Mwanamsangu, Maligo Katebalila, Amani Shao, John Changalucha, Soori Nnko, Mwita Wambura

**Affiliations:** 1 Department of Sexual and Reproductive Health, National Institute for Medical Research, Mwanza Centre, P.O Box 1462, Mwanza, Tanzania; 2 Sauti Project, Jhpiego Tanzania - an affiliate of Johns Hopkins University, Dar es Salaam, Tanzania

**Keywords:** Anxiety, Depression, PHQ-4, Reliability, Validity, Adolescent girls and young women, Tanzania

## Abstract

**Background:**

Literature suggests that most mental disorders have their onset in childhood and adolescence, but go undiagnosed until adulthood. Shorter versions of the screening tools such as the Patient Health Questionnaire with four items (PHQ-4) may help to improve screening coverage. This study assessed the psychometric properties of the PHQ-4 in screening for core symptoms of depression and anxiety among out of school adolescent girls and young women (AGYW).

**Methods:**

This is a cross-sectional analysis of data from a cluster randomized controlled trial conducted among AGYW between June and July 2018 in North-West Tanzania. Two thousand four hundred twenty-six out-of-school AGYW aged 15 to 23 years were included. Data were collected on tablets using audio computer-assisted self-interviews (ACASI). Cronbach’s α was used to measure the reliability of the PHQ-4 while confirmatory factor analysis (CFA) and principal components analysis (PCA) were used for construct validity assessment. In CFA, three criteria were used to assess how well the model fits the data: Standardized Root Mean Square Residual (SRMR), the Comparative Fit Index (CFI), the Root Mean Square Error of Approximation (RMSEA) and 90% confidence interval for RMSEA.

**Results:**

Of the 2426 participants, 33.8 and 35.5% screened positive for core symptoms of anxiety (GAD-2 ≥ 3) and depression (PHQ-2 ≥ 3), respectively. Cronbach’s α of the PHQ-4 was 0.81. Both items-correlation and corrected items-correlation of the PHQ-4 had total correlations above 0.5 (*p* < 0.01). CFA showed that all items loaded significantly onto the single factor, and loadings were strong, ranging from 0.67 to 0.77 (*p* < 0.01). CFA indicates that the PHQ-4 scale stand for a unidimensional construct with good model fit (CFI = 0.995, SRMR = 0.013, RMSEA = 0.054 and 90% CI for RMSEA (0.031–0.079)). PCA confirmed two distinct components; GAD-2 (anxiety) and PHQ-2 (depression). Those who reported having suicidal thoughts and social function problems had significantly higher scores on PHQ-2, GAD-2, and PHQ-4 screening items (*p* < 0.01).

**Conclusions:**

The findings suggest that the PHQ-4 scale can reliably and validly screen for core symptoms of depression and anxiety among out of school AGYW. This tool is short and easy to administer. Thus, the PHQ-4 scale can be very useful in screening for anxiety and depression symptoms in the community, primary health facilities, research and programmatic settings.

## Background

Depression and anxiety are the leading causes of disability, morbidity, and mortality among adolescent girls and young women (AGYW) aged 15 to 29 years in Africa [1]. Evidence increasingly shows varied prevalence across different populations including AGYW and people living with HIV (PLHIV) [2, 3]. For instance, a study conducted in Kenya and Zambia using the four-item Patient Health Questionnaire (PHQ-4) reported the prevalence of moderate and severe symptoms of anxiety and depression of 3.7 and 9.4% respectively, among AGYW [2]. A study conducted in Tanzania reported a prevalence of depression of 27% among children and adolescents living with HIV [3].

Literature suggests that most mental disorders go undiagnosed until adulthood, even though their onset is around childhood and adolescence [4–6]. Factors related to access to mental health services contribute to this gap. These include individual factors such as lack of awareness, societal factors, including myths regarding mental health disorders and systemic factors, for example, the centralization of services and inadequately qualified staff [7, 8]. Consequently, AGYW with anxiety and depression continue to experience functional impairment, academic difficulties, suicidal ideation and attempts, and further risk of the futile cycle of mental illnesses [9–12]. Moreover, children born by mothers with these conditions may also suffer suboptimal cognitive development [13].

Several recommendations have been put forward in the literature on how to improve access and utilization of mental health services. These recommendations include decentralization and task shifting in the provision of mental health services, to maximize uptake of services both at lower-level facilities and the community [8, 14]. To be able to realize this, screening scales must be made available to enable identification of probable mental health disorder patients and provide referral to health facilities for diagnosis. Scales such as Patient Health Questionnaire-9 (PHQ-9) and Generalized Anxiety Disorders-7 (GAD-7) have been used for depression and anxiety, respectively. However, to improve efficiency during mass screening or in busy outpatient clinics, shorter versions of the scales have been researched and successfully validated among clinic-based and general populations [15, 16]. One of the shorter versions of the screening scales is the Patient Health Questionnaire-4 (PHQ-4) [9]. This is a four-item screening tool for both depression and anxiety [9]. This tool was validated in the general adult population in Germany [16] and among college students in the United States [17]. A study conducted among college students in the United States demonstrated the PHQ-4 scale to be an effective tool for screening depression and anxiety, with the area under the curve (AUC) of 0.835 and 0.787 for depression and anxiety respectively [17]. While validation studies have been conducted in other settings [16, 17], findings from these studies cannot be generalized to Tanzania context, given the cultural and study population differences (out of school AGYW in Tanzania versus college students in the USA [17] and general adult population in Germany [16]). Besides, there are also differences in survey administration methods where the present study uses audio computer-assisted self-interviews (ACASI) while previous studies used interviewer administered techniques [16, 17]. Therefore, this study aims to explore the reliability and validity of the PHQ-4 in a large out-of-school AGYW population in Tanzania using ACASI data collection technique.

## Methods

This study uses data from a cluster randomized controlled trial, CARE study, conducted in Kahama, Ushetu and Msalala districts. CARE study was approved by the Medical Research Coordination Committee of the National Institute for Medical Research (NIMR/HQ/R.8a/Vol.IX/2287) and the Johns Hopkins University IRB (00007976). CARE study is also registered at ClinicalTrials.gov, number NCT03597243. Details on CARE study are documented elsewhere [18].

### Study population

Eligible for the study were AGYW aged 15 to 23 years who were out of school, residents of the village selected for study participation and who have completed 10 h of social and behavior change communication (SBCC) sessions. Besides, AGYW were eligible if they provided voluntary informed consent to take part in the study. The current study utilizes data from the first follow-up round of CARE study (*n* = 2426). Participants were enrolled from 30 villages from June to July 2018. The authors used second-round data because the data consists of additional information on mental health disorders, including the effect of depression and anxiety symptoms on daily activities and social relationships, and suicidal ideation.

### Procedures

During SBCC sessions, potential participants were informed about the dates and locations of CARE study recruitment. Thus, the potential participants were able to reach the recruitment site for pre-screening consent and eligibility screening at baseline until the sample required was reached. During follow up rounds, participants were also informed of data collection dates and locations in their SBCC groups.

Within the CARE study, the study participants were interviewed using audio computer-assisted self-interviews (ACASI). ACASI is an electronic self-administered questionnaire, where participants listen privately to pre-recorded interview questions using a tablet connected to earphones [18, 19]. The participants could also simultaneously read each interview question, including the PHQ-4 items, on the tablet screen as the question was being read. The interviews were conducted in settings within the community, which ensured the privacy of study participants. Data collection was facilitated by research assistants who were trained on research ethics, interviewing skills, data management as well as study-specific procedures. The research assistants also received online training in good clinical practice (GCP).

### Instrument and measures

The ACASI questionnaire included questions on demographics and mental health symptoms (S1 Questionnaire). Mental health screening was conducted using the 4-item PHQ for depression and anxiety developed by Kroenke and colleagues [9].

The PHQ-4 is a concise tool composed of the PHQ-2 screening tool for depression and generalized anxiety disorders (GAD) screener (GAD-2) [9]. PHQ-2 collects self-reports of two core symptoms of depression according to the Diagnostic and Statistical Manual of Mental Disorders, Fourth Edition (DSM-IV) [9]. The depression questions were phrased as: “Over the *last 2 weeks,* how often have you been bothered by the following problems?”; ‘Feeling down, depressed, or hopeless’ and ‘Little interest or pleasure in doing things’ [9]. While GAD-2 collects two core symptoms of anxiety and was phrased as “Over the *last 2 weeks,* how often have you been bothered by the following problems?”; ‘Feeling nervous or anxious or on edge’ and ‘Not being able to stop or control worrying’ [9]. Participants responded using a 4-point Likert-type of options; ‘not at all’ =0, ‘several days but less than one week’ =1, ‘more than half the days’ =2, ‘nearly every day’ =3. Positive screening for anxiety was assigned for participants if the score of the two core symptoms of anxiety was greater than or equal to three (GAD-2 ≥ 3) [9]. Similarly, for the two core symptoms of depression, scores of greater than or equal to three (PHQ-2 ≥ 3) were assigned positive screening [9]. Lastly, the overall measure of anxiety and depression was graded as normal (0–2), mild (3–5), moderate (6–8), and severe (9–12) [9].

Two additional questions were also obtained from the Patient Health Questionnaire with nine items (PHQ-9) [20]. The PHQ-9 questions were worded as: ‘If you checked off *any* problems*,* how *difficult* have these problems made it for you to do your work, take care of things at home, or get along with other people?’ and ‘Have you had thoughts that you would be better off dead or of hurting yourself in some way in the last two weeks’ [20]. All PHQ-4 data for the current study were collected as baseline assessments during the second round of data collection.

### Data management

Data from ACASI were collected using tablets and sent to the server located at the National Institute for Medical Research (NIMR) in Mwanza daily through a secure file transfer protocol. The data were extracted by the data manager for data quality checks. While the data collectors were still in the field, data queries were generated and sent to the field for resolution. The process continued until all the queries were solved, and then the final datasets were created (S1 Dataset).

### Data analysis

Data were managed and analysed using SAS 9.4 (SAS Institute Inc.; Cary, North Carolina) and STATA 14 (College Station, TX: StataCorp LP). Descriptive statistics (frequencies, means, and standard deviations) were used to describe the AGYW in terms of their demographic characteristics. An item analysis was conducted to assess score distributions, missing data patterns and floor/ceiling effects. Floor or ceiling effects were considered to be present if more than 15% of participants attained the lowest or highest possible scores, respectively [21, 22]. Analysis of score distribution was based on descriptive statistics for ordinal data. Item characteristics of the PHQ-4 items (item-means, item-intercorrelation and corrected item-total correlations) were explored.

For reliability, the internal consistency of the PHQ-4 was assessed. Cronbach’s alpha was used to measure internal consistency (i.e. the extent to which all items on a scale were inter-related or contribute positively towards measuring the same construct) [23–25]. Most of the time, the acceptable range of Cronbach’s alpha is a value of 0.70 or above [26]. However, it depends on how a measure is being applied and has been suggested that in research more stringent cut-offs (i.e. 0.80 or higher and 0.90 or higher) should be used [27]. Due to the ordinal nature of the PHQ-4 items, the estimation of the Cronbach’s alpha was based on the polychoric correlation matrix [28].

To explore whether the four items of the scale depicted a single dimension, confirmatory factor analysis (CFA) was used to assess model fit for a one-factor solution. Considering the ordinal nature of the data into account, the analysis was based on the two-stage estimation approach [29]. In stage one, the polychoric correlation was computed. Then, the model was analyzed with the appropriate weight matrix computed in stage one. This procedure ensures unbiased parameter estimates and standard error for ordinal data [29]. To get more accurate information regarding the goodness of model fit, Standardized Root Mean Square Residual (SRMR), the Comparative Fit Index (CFI), the Root Mean Square Error of Approximation (RMSEA) and 90% confidence interval for RMSEA were used [30]. CFI values greater than 0.94 indicate good fit [31], RMSEA values less than 0.09 indicate a fair or adequate error of approximation whereas values less than 0.055 indicate a small error [31–33] and RMSEA = 0 indicate exact model fit of approximation [34]. Likewise to the RMSEA, SRMR values less than 0.09 indicate fair or adequate fit, whereas SRMR values less than 0 .055 indicate a good fit [31].

Principal components analysis (PCA) using a varimax-rotated component-matrix for the four screening items (two items each for GAD-2 and PHQ-2) were also used for construct validity assessment. In principal components analysis, the observed variables are transformed into a smaller number of artificial variables (i.e. principal components), which will account for most of the variance in the observed variables. The principal components can be used as a criterion variable in the consequent analysis [35, 36]. A varimax rotation (orthogonal rotation) method was used with the assumption that there is no correlation between components [9, 16, 37]. Furthermore, the association was checked between the PHQ-2, GAD-2, and PHQ-4 scores for respondents who had reported suicidal thoughts or social dysfunction versus those who had not reported suicidal thoughts or social dysfunction in the past 2 weeks.

## Results

### Participants’ characteristics

A total of 2426 participants were interviewed. The mean age of participants was 20 years (standard deviation (SD) = 2.5), with 1393 (57%) being 20 to 23 years old. Over half of AGYW had a primary education (56%), 1224 married (51%) and 1550 had children (64%). The proportion of participants with positive screening for anxiety (GAD-2 ≥ 3) was 33.8%, and depression (PHQ-2 ≥ 3) was 35.5% (Table 1).

**Table 1:** Demographic Characteristics of Participants

	***n*** = 2426	%
	Mean (±SD)	
**Age (Years)**	19.7 (2.5)	
**Age (Years)**		
15–19	1033	42.6
20–23	1393	57.4
**Education level**		
No formal/Incomplete primary education	583	24.0
Primary school	1364	56.2
Complete/incomplete secondary school	479	19.7
**Marital status**		
Single	1039	42.8
Married (polygamous/monogamous/cohabiting)	1224	50.5
Formerly-married (separated/divorced/widowed)	163	6.7
**Family composition (Lives with)**		
Alone/ younger siblings/employer	51	2.1
Husband	1187	48.9
Parents/relative/ elder siblings	1188	49.0
**Ever had children**		
Yes	1550	63.9
No	876	36.1
**Living in TASAF households**^**a**^		
Yes	212	8.7
No	2214	91.3
**Ever went to bed hungry due to lack of food**^**b**^		
Yes	298	12.3
No	2128	87.7
**Anxiety symptoms (GAD-2** ≥ 3**)**	821	33.8
**Depression symptoms (PHQ-2** ≥ 3**)**	861	35.5

^**a**^Reported living in a household supported by a social welfare program; Tanzania Social Action Fund (TASAF); ^**b**^Reported staying a day or going to bed hungry due to lack of food within the last 4 weeks

### Item characteristics and reliability

Descriptive statistics of the individual items for depression and anxiety screening sub-scale (i.e. PHQ-2 and GAD-2) and the total scale (i.e. PHQ-4) are shown in Table 2. The average anxiety score was 1.79, and the SD was 1.71 while for depression the average score was 1.96, and the SD was 1.66. The score distribution of the lowest response (i.e. not at all) ranged between 46.0 and 47.8% of the items in anxiety and between 35.0 and 48.2% for depression. No ceiling effects observed; all items showed floor effects. Both items-correlation and corrected items-correlation have total correlations above 0.5. All of these correlations were statistically significant at *p* < 0.01.

**Table 2:** Item scores and scale correlations for study participants

Over the last two weeks, how often have you been bothered by the following problems	Mean (SD)	Score distribution %				^a^Item–total correlation	^b^Corrected item-total correlation
		0	1	2	3		
**Anxiety screener (GAD-2) sum score**	1.79 (1.71)						
1. Feeling nervous, anxious or on edge	0.92 (1.01)	**46.00**	25.06	19.54	9.40	0.76	0.64
2. Not being able to stop or control worrying	0.87 (0.98)	**47.82**	26.01	17.97	8.20	0.76	0.64
**Depression screener (PHQ-2) sum score**	1.96 (1.66)						
3. Little interest or pleasure in doing things	0.85 (0.97)	**48.15**	26.05	18.55	7.25	0.73	0.60
4. Feeling down, depressed or hopeless	1.11 (1.01)	**35.04**	30.87	22.42	11.67	0.74	0.59
**Total score (PHQ4)**	3.75 (2.98)						

Scale items scored 0, 1, 2, 3 based on responses provided (not at all, several days, more than half the days, and nearly every day).

^**a**^Correlation between the item and the total score from the scale (i.e. the item itself is included in the total); ^**b**^Correlation between the item and the overall score from the scale (i.e. the item itself is not included in the total)

The Cronbach’s alpha for PHQ-4, GAD-2, and PHQ-2 computed for internal-consistency (i.e. reliability), were α = 0.81, α = 0.74, and α = 0.65 respectively. None of the items if deleted improves reliability (i.e. increases Cronbach’s alpha) (Table 3).

**Table 3:** Item Internal consistency of the scales

Over the last 2 weeks, how often have you been bothered by the following problems			
	^**a**^4-Item alpha	2-Item alpha	^**b**^alpha if item- dropped
**Anxiety (GAD-2)**			
1. Feeling nervous, anxious or on edge	0.81	0.74	0.75
2. Not being able to stop or control worrying			0.74
**Depression (PHQ-2)**			0.77
3. Little interest or pleasure in doing things		0.65	0.77
4. Feeling down, depressed or hopeless			

^**a**^The overall alpha (Cronbach’s α: Indicates the internal reliability of the four items (conducted with the entire sample)); ^**b**^Values of the overall alpha (Cronbach’s) if that item isn’t included in the calculation

### Construct validity

CFA results showed that all items loaded significantly onto the single factor, and loadings were strong, ranging from 0.67 to 0.77 (p < 0.01). CFA indicate that the PHQ-4 scale stand for a unidimensional construct with good model fit (CFI = 0.995, SRMR = 0.013, RMSEA = 0.054 and 90% CI for RMSEA (0.031–0.079)) (Table 4). Principal components analysis (PCA) with the varimax-rotated component-matrix for the four screening items (i.e. two items each for PHQ-2 and GAD-2) shows that the two components explained 82% of the total variance. Two anxiety screening items had the highest loading on component 1, and the two depression screening items had the highest loading on component 2 (Table 4). Besides, the association was checked between PHQ-2, GAD-2, and PHQ-4 scores for individuals who had reported social function problem and suicidal thoughts versus those who had not in the past 2 weeks (Table 5). Those who reported having suicidal thoughts and social function difficulties had significantly higher scores on PHQ-2, GAD-2, and PHQ-4 screening items (*p* < 0.01).

**Table 4:** Principal Components Analysis and confirmatory factor analysis (CFA) for PHQ-4. (Factorial Validity)

Four-items of anxiety and depression scale	Component loadings		Goodness-of-fit statistics
	^**a**^Component1	^**b**^Component2	Confirmatory factor analysis
1. Feeling nervous, anxious or on edge	**0.834**	0.230	CFI = 0.995
2. Not being able to stop or control worrying	**0.799**	0.272	SRMR = 0.013
3. Little interest or pleasure in doing things	0.333	**0.716**	RMSEA = 0.054
4. Feeling down, depressed or hopeless	0.186	**0.865**	90%CI for RMSEA (0.031,0.079)

Note: Extraction method: principal components analysis. Rotation method: varimax;^a^ Items on anxiety (GAD-2);

^**b**^Items on depression (PHQ-2); Components accounted for 82% of the variance

**Table 5:** Comparison of Mean Scores on Scales between Groups that have Depression and/ or Anxiety and reported suicidal thoughts and social function

	Two-item anxiety	Two-item depression	Four-item scale
	scale score (GAD-2)	scale score (PHQ-2)	score (PHQ-4)
	Mean(±SE)	Mean(±SE)	Mean(±SE)
^**a**^Social function difficulties			
Yes	2.60 (0.04)	2.78 (0.04)	5.39 (0.07)
No	1.48 (0.06)	1.75 (0.05)	3.23 (0.09)
^**b**^Suicidal thoughts			
Yes	2.99 (0.06)	3.13 (0.06)	6.12 (0.09)
No	1.82 (0.04)	2.05 (0.04)	3.87 (0.07)

^**a**^How difficult have these problems (anxiety and/ or depression) made it for you to do your work, take care of things at home, or get along with other people? (No = Not difficult at all, yes = somewhat difficult /very difficult/extremely difficult); Scores for individuals with social function problem were significantly higher compared to those who did not have (*p* < 0.01)

^**b**^ Over the last 2 weeks, how often have you had thoughts that you would be better off dead or of hurting yourself in some way? (No = Not at all, yes = several days/more than half the days/nearly every day); Scores for individuals with suicidal thoughts were significantly higher compared to those who did not have (*p* < 0.01)

## Discussion

This study aimed to assess the psychometric properties of PHQ-4 among out-of-school AGYW in Tanzania. We evaluated the reliability and validity of anxiety and depression screening scale (PHQ-4) among out of school AGYW. The findings from this study suggest that PHQ-4 can reliably and validly screen for core symptoms of depression and anxiety among AGYW. Results from CFA indicate that the PHQ-4 is a structurally valid, unidimensional measure of anxiety and depression with all items loaded significantly into a single factor and loadings were ranging from 0.67 to 0.77. Principal component analysis with varimax-rotated component matrix ratified the original allocation of the items to the PHQ-4 scales, with the two anxiety items having the maximum factor loadings on component 1 and the two depression items having the maximum factor loadings on component 2. Furthermore, those who reported having suicidal thoughts and social function difficulties had statistically significantly higher scores on PHQ-2, GAD-2, and PHQ-4 screening items (*p* < 0.01) than the others. Similar results were observed from a study conducted among primary-care patients in the United States and college students at a Midwestern university [9, 17].

The questionnaire was observed to be measuring a single construct, with all four questions having corrected item-total correlations above 0.3, which is the normally accepted cut-off level for keeping or discarding questionnaire items [38, 39]. Our findings were similar to a study on psychometric properties of PHQ-4 conducted among college students, although the correlation obtained was a bit higher compared to the current study [17]. In the current study, the internal consistency of PHQ-4 was supported by Cronbach’s alpha (α = 0.81), which was above 0.70. However, for PHQ-2 and GAD-2 the Cronbach’s alpha were a bit lower: α = 0.65, α = 0.74, respectively. These findings were consistent but varied slightly from a study by Khubchandani et al. [17], who measured psychometric properties of PHQ-4 among college students (α = 0.81, α =0.76, and α = 0.82 for PHQ-4, PHQ-2 and GAD-2 respectively) and a study conducted by Lowe et al. among the general population in German (α = 0.78, α = 0.75, and α = 0.82 for PQH-4, PHQ-2 and GAD-2 respectively) [16]. The slight variations in the Cronbach’s alpha between the current study and other previous validation studies conducted in Germany and USA [16, 17] may be caused by the difference in sample characteristics, setting and survey administration methods. However, all the values obtained by the current study are within acceptable reliability range [26].

The item response distributions were skewed to the lower scores, and floor effects were observed for all items. This means the present study sample response distribution is skewed towards no symptoms of anxiety or depression. The results of the floor effect could impair the impact of responsiveness and sensitivity [39, 40]. However, all of the alternative options in each item from the present study were selected, which point out that all response options were pertinent. Similar results were observed from a related study conducted using the Hospital Anxiety and Depression Scale (HADS) [41]. Besides, this study was based on data from the out-of-school AGYW population where a moderate proportion was shown to have symptoms of anxiety and depression. Thus, this problem might be related to the sample rather than the instrument.

### Strengths and weaknesses

To our knowledge, this is the first study to assess the psychometric properties of the PHQ-4 scale in a large sample of out of school AGYW using ACASI. However, the scale has already been tested in other settings among samples with different characteristics [9, 17]. The current study sample was drawn from the community, unlike other studies where samples were drawn from healthcare facilities, clinics or the general adult population [9, 16].

This study has some limitations; the results are unlikely to be generalized to all AGYW in Tanzania since the sample was restricted to out of school AGYW from three districts in Shinyanga region in North West of Tanzania. Also, no clinical diagnostic evaluation was conducted as a reference standard. Thus, we cannot infer measures of screening accuracy, such as sensitivity and specificity for the PHQ-4. In addition, the screening diagnosis using PHQ-4 can have false-positive results since no strategies for confirming the diagnosis were used. This would indicate high levels of anxiety and depression without clinical problems [42, 43]. Furthermore, the current study drew data from a longitudinal study (CARE Study), in which the period between study data collection rounds was 6 months, therefore test-retest reliability could not be done. Nevertheless, the current study showed that the participants who screened positive for anxiety and depression were significantly more likely to report suicidal ideation and challenges with social interactions and functioning. Thus, the PHQ-4 can effectively be used for screening core symptoms of depression and anxiety, and positive screens can be referred to the health facility for diagnosis and management as appropriate.

### Implications

AGYW who are out of school experience relatively more psychological distress compared to their school-attending peers [44]. As stated earlier, most mental health disorders are undiagnosed until adulthood [9–11]. As a result, the developmental potential, social life and economic impact of the AGYW are compromised [9–12]. This highlights the need for improving the screening and management of mental health disorders.

Utilizing a community sample, our assessment of PHQ-4 with out-of-school AGYW indicates that this tool can be used as a rapid, psychometrically sound and effective screening tool for depression and anxiety symptoms. The PHQ-4 is easy to administer, and the scoring system can easily be implemented through on-the-job training. Literature suggests that the decentralization of mental health services has the potential to expand coverage [8]. The tools such as the PHQ-4 can, therefore, be very useful in screening for anxiety and depression in the community, primary health facilities, research and programmatic settings [2, 9, 16]. This can be coupled with referral to higher-level facilities for diagnosis and management for those who screen positive.

## Conclusions

The findings from this study suggest that PHQ-4 item measure can reliably and validly measure core symptoms of depression and anxiety among the out of school AGYW. Overall, the PHQ-4 reliability and validity were reasonable and observed to be psychometrically sufficient and helpful for the measurement of anxiety and depression symptoms among AGYW. However, the problem with floor effects was observed for all items, although no serious impact on the factor structure observed due to this problem. But users need to be aware, as this problem could impair the impact of responsiveness and sensitivity. In spite of this, PHQ-4 can be endorsed to assess anxiety and depression symptoms among AGYW.

## Supplementary information

**Additional file 1.** Questionnaire

**Additional file 2.** Minimal dataset

## Data Availability

All data underlying the findings reported in this manuscript are provided as supporting information for this manuscript.

## Abbreviations

ACASI: Audio computer assisted self-interviews
AGYW: Adolescent girls and young women
AUC: Area under the curve
CARE: Cash Transfer to Adolescent Girls and Young Women to reduce sexual risk behaviour
CFA: Confirmatory factor analysis
CFI: Comparative Fit Index
DSM-IV: Diagnostic and Statistical Manual of Mental Disorders, Fourth Edition
GAD-2: Generalized Anxiety Disorders-2
GAD-7: Generalized Anxiety Disorders-7
GCP: Good clinical practice
HIV: Human Immunodeficiency Virus
IRB: Institutional Review Board
NIMR: National Institute for Medical Research
PCA: Principal components analysis
PHQ-2: Patient Health Questionnaire-2
PHQ-4: Patient Health Questionnaire-4
PHQ-9: Patient Health Questionnaire-9
PLHIV: People living with human immunodeficiency viruses
RMSEA: Root Mean Square Error of Approximation
SBCC: Social and behavior change communication
SD: Standard deviation
SRMR: Standardized Root Mean Square Residual
