# Supplementary material for: The psychometric properties of PHQ-4 anxiety and depression screening scale among out of school adolescent girls and young women in Tanzania: a cross-sectional study
Source: BMC Psychiatry. 2020 Jun 19;20:321. doi: 10.1186/s12888-020-02735-5 (PMC7304148; doi:10.1186/s12888-020-02735-5)
Supplement: Supplementary file 1 — Additional file 1. Questionnaire [file 12888_2020_2735_MOESM1_ESM.docx]

**Questionnaire**

Participant ID: ____________________

**Questionnaire**

1. When were you born? |__|__|-|__|__|-|__|__|__|__|
2. What is your education level?
3. I have no formal education
4. Incomplete primary school education
5. Completed primary school education
6. Incomplete secondary school education
7. Completed secondary school education
8. Marital status
9. Single
10. Married (monogamous marriage)
11. Married (Polygamous marriage)
12. Cohabiting
13. Divorced/Separated
14. Widow
15. Who do you live with?
16. I live with my parents
17. I live with my relatives/friends
18. I live with my elder siblings
19. I live with my younger siblings
20. I live with my husband
21. I live alone
22. Other; specify______
23. Is your household receiving support from the Tanzania Social Action Fund (TASAF)?
24. Yes
25. No
26. In the last four weeks, have you ever stayed or slept hungry due to lack of food?
27. Yes
28. No
29. Do you have children?
    1. Yes
    2. No
30. Patient Health Questionnaire-4 (PHQ-4) and additional questions from PHQ-9

**Over the last 2 weeks, how often have you been bothered by the following problems?**

- 1. Feeling nervous, anxious or on edge

0. Not at all

1. Several days

2. More than half the days

3. Nearly every day

2. Not being able to stop or control worrying

0. Not at all

1. Several days

2. More than half the days

3. Nearly every day

3. Little interest or pleasure in doing things

0. Not at all

1. Several days

2. More than half the days

3. Nearly every day

4. Feeling down, depressed, or hopeless

0. Not at all

1. Several days

2. More than half the days

3. Nearly every day

5. If you checked off any problems on PHQ-4 questions above*,* how difficult have these problems made it for you to do your work, take care of things at home, or get along with other people?

0. Not difficult at all

1. Somewhat difficult

2. Very difficult

3. Extremely difficult

6. Have you had thoughts that you would be better off dead or of hurting yourself in some way in the last two weeks

0. Not difficult at all

1. Somewhat difficult

2. Very difficult

3. Extremely difficult
